# Predicting drug‐perturbed transcriptional responses using multi‐conditional diffusion transformer

**DOI:** 10.1002/qub2.70016

**Published:** 2025-09-21

**Authors:** Qifan Hu, Zeyu Chen, Jin Gu

**Affiliations:** ^1^ MOE Key Laboratory of Bioinformatics BNRIST Bioinformatics Division Department of Automation Tsinghua University Beijing China

**Keywords:** diffusion model, perturbation, transcriptome

## Abstract

Drug‐perturbed transcriptomes are important for personalized medicine and drug discovery. Nevertheless, the existing high‐throughput screening and sequencing techniques for drug‐perturbed transcriptomes remain expensive and time‐consuming. In this study, we propose a novel multi‐condition diffusion transformer model, designated as perturbation diffusion transformer (PertDiT), which is tailored for conditionally generating the perturbed transcriptomes based on drug text information. PertDiT combines the potent transformer architecture with the text representation of pre‐trained large language models and utilizes a novel perturbation and transcriptome fusion modules. We have designed two network structures, namely, CrossDiT and CatCrossDiT, applicable to drug discovery and personalized medicine scenarios, respectively. Through a comprehensive set of metrics and an effective data splitting strategy, our model outperforms existing methods, demonstrating a superior ability in post‐perturbation transcriptome reconstruction and the prediction of perturbation‐induced transcriptional changes. The rationality and effectiveness of the model structure have also been meticulously validated.

## 1 INTRODUCTION

Drug‐perturbed transcriptomes can comprehensively depict the mechanism of action of drugs and play a significant role in the fields of personalized medicine [1, 2] and new drug discovery [3]. Existing high‐throughput sequencing technologies can enable large‐scale drug‐perturbed transcriptome experiments, such as sci‐Plex3 [4] and L1000 [5]. However, exclusive reliance on sequencing experiments entails high time and costs, rendering it arduous to encompass the extensive combinatorial space of perturbation numbers and complex cellular states. Therefore, utilizing algorithms and public datasets for in‐silico perturbed transcriptome prediction is preferred.

Datasets used for perturbed transcriptome prediction contain the pre‐ and post‐perturbation states, as well as different perturbation information. Existing methods basically adopt deep generative model architectures. For example, scGen [6] based on variational autoencoder directly calculates the variation between the control and perturbed states and applied for new condition states; biolord [7] and ChemCPA [8] based on disentanglement can achieve counterfactual inference through extracting latent space states that do not contain additional drugs or other covariates and combining them with different new conditions. The recently published PRnet [9] adopts an encoder–decoder structure similar to conditional‐VAE and combines effective perturbation modeling to achieve efficient and accurate post‐perturbation transcriptome prediction. The drug recommendation framework and perturbation atlas developed by it can also be applied to more scenarios of drug discovery and drug screening.

With the development of multimodal and artificial intelligence generated content technologies, diffusion models have achieved state‐of‐the‐art (SOTA) performance in multiple fields such as images, videos, and audio [10]. The high‐quality generation of diffusion models and the multimodal feature fusion and extraction capabilities of cross‐attention and self‐attention modules in the transformer architecture are applicable to most data generation tasks [11, 12, 13, 14]. Existing methods have already applied diffusion to spatial transcriptome data [15] and single‐cell data [16]. Therefore, the application of diffusion models and the transformer architecture in the field of perturbed transcriptome generation is quite promising.

In this study, we introduce a new multi‐condition diffusion transformer model, which is tailored for predicting perturbed bulk transcriptomes (gene expression profiles), namely, perturbation diffusion transformer (PertDiT). Our method combines the powerful transformer architecture with the text representation of pre‐trained large language models and utilizes innovative perturbation and transcriptome fusion modules to fuse the pre‐perturbed transcriptome and perturbation information, respectively, for one‐to‐one post‐perturbed transcriptome prediction. We design two network structures, namely, CrossDiT and CatCrossDiT, for the scenarios of new drug discovery and personalized medicine, respectively. Through well‐designed 10 comprehensive metrics and an effective data splitting strategy, we verify the effectiveness of the two structures. The main contributions of this paper are as follows:

- We propose for the first time a multi‐condition diffusion model for predicting post‐perturbation transcriptomes from pre‐perturbation states in a one‐to‐one manner.
- We present an innovative text‐based representation method for chemical perturbations and doses.
- We provide comprehensive evaluation metrics and an efficient data splitting benchmark.

## 2 RESULTS

**FIGURE 1:**
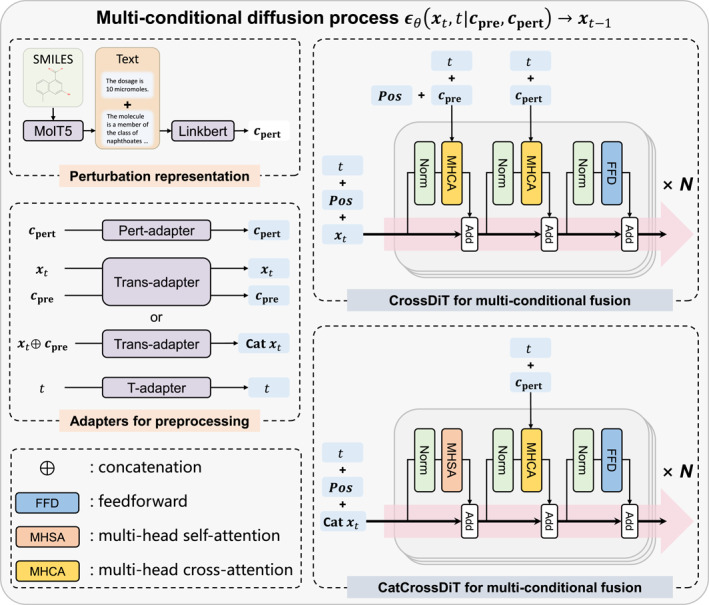
Overview of the proposed perturbation diffusion transformer (PertDiT) model.

Figure 1 presents the overview of PertDiT structure, a one‐to‐one drug‐perturbed transcriptome generator. PertDiT employs the training and sampling processes of the multi‐conditional denoising diffusion probabilistic models (DDPM) to control the generation of the perturbed transcriptome ***x***_0_ using pre‐perturbed condition ***c***_pre_ and perturbation condition ***c***_pert_.

To enhance the extensibility of perturbation modeling, inspired by the concept of BioTranslator [17], we propose a unified chemical perturbation representation approach based on text embeddings. Specifically, the simplified molecular input line entry system (SMILES) [18] of the perturbation condition is represented by text descriptions generated by the pre‐trained MolT5 [19]. Subsequently, the dosage prompt is added, and the resulting text is converted to text embeddings using the pre‐trained Linkbert [20].

The multi‐conditional DDPM noise predictor ***ϵ***_*θ*_(***x***_*t*_,*t*| ***c***_pre_, ***c***_pert_) primarily comprises three adapters and transformer layers with multiple inputs, including the noisy data ***x***_*t*_, conditions ***c***_pert_ and ***c***_pre_, and timestep *t*. Before fed into the transformer layers, each of them undergoes preprocessing from different adapters. Specifically,

- Timestep adapter (T‐adapter): *t* is converted to sine‐cosine encoding and processed by a multilayer perceptron (MLP) to generate the timestep embedding;
- Transcriptome adapter (Trans‐adapter): ***x***_*t*_ and ***c***_pre_ are processed by MLP and added learnable gene representations as special positional embeddings (**Pos**) in conjunction with the timestep embedding;
- Perturbation adapter (Pert‐adapter): ***c***_pert_ is processed by MLP and added timestep embedding.

Two types of transformer layers are designed, namely, CrossDiT and CatCrossDiT (Figure 1), which are based on different multi‐conditional fusion strategies. CrossDiT fuses the pre‐perturbed condition and perturbation conditions, respectively, through two cross‐attention modules. CatCrossDiT fuses the pre‐perturbed condition by concatenating the noisy data and using the self‐attention, subsequently fusing the perturbation condition using cross‐attention.

### 2.1 Comparative analysis

In this study, we compare our method with the most recent SOTA method PRnet and the classical method ChemCPA. To efficiently and comprehensively evaluate the model’s performance, we propose a novel dataset splitting strategy. This strategy divides the whole dataset into a training set, a validation set, and three test sets: Drug_unseen, Cell_line_unseen, and Both_unseen (Figure S1). This splitting strategy enables assessment of the generalization ability of models on unseen drugs, unseen cell lines, and both unseen factors based on a single training. We design 10 different metrics (methods), which evaluate the effect of transcriptome reconstruction (*R* ^2^) and the prediction of gene expression changes (PCC(ln **FC**)), in combination with different levels of grouping granularity, ranging from fine to coarse (per_sample to drug).

**FIGURE 2:**
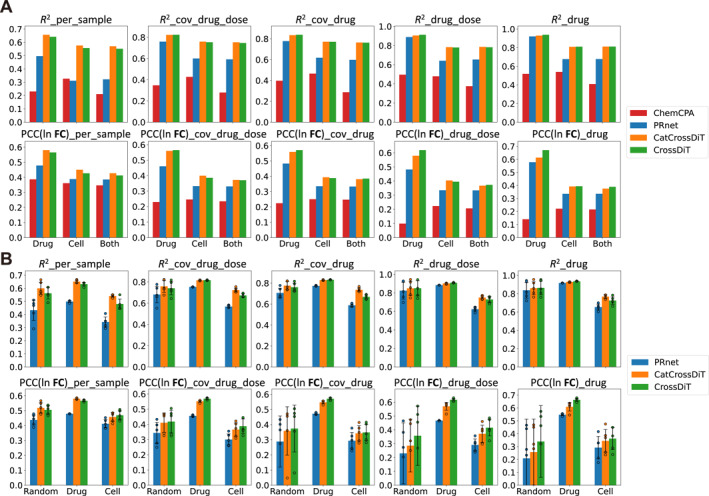
Performance of out‐of‐distribution post‐perturbation transcriptome prediction. (A) *R* ^2^ (top row) and PCC(ln **FC**) (bottom row) are displayed by bar plots and the grouping granularity ranges from fine to coarse from left to right. Each metric corresponds to three different splits under our proposed strategy: unseen drugs (Drug), unseen cell lines (Cell) and both unseen (Both), which are used to test the generalization ability of the model on unseen drugs, unseen cell lines and both unseen, respectively. (B) Ten metrics are shown by bar plots. Each metric corresponds to three different splitting strategies used in PRnet: random (Random), unseen drugs (Drug), and unseen cell lines (Cell). Each metric in each split of each method is presented as the mean value (bar) ± SD (error bar) and five data points.

As shown in Figure 2A, our proposed two model structures outperform PRnet and ChemCPA across all splits and metrics, indicating that the utilization of a multi‐conditional diffusion model and transformer structure can effectively model perturbations and achieve more accurate post‐perturbation transcriptome reconstruction and prediction of perturbation‐induced transcriptional changes. The overall performance of CatCrossDiT and CrossDiT is comparable, and due to the multi‐conditional model, there exists a trade‐off between the modeling effects of the two conditions. CatCrossDiT exhibits superior fusion capabilities of transcriptome information, leading to enhanced efficacy in unseen cell line splitting scenarios and finer‐grained grouping metrics. Conversely, CrossDiT exhibits improved fusion of perturbation information, leading to increased effectiveness in unseen drugs and coarser grouping metrics, attributable to its superior capture of the consistent effect of perturbation information. In the novel Both_unseen grouping, the two methods exhibit comparable performance. Furthermore, CatCrossDiT demonstrates superior transcriptome reconstruction due to its focus on transcriptome modeling, whereas CrossDiT excels in predicting perturbation‐induced changes owing to its emphasis on perturbation fusion.

For a fair comparison, we additionally use the five‐fold cross‐validation and three out‐of‐distribution splits: random, unseen drugs and unseen cell lines, same as in PRnet. We only compare our method with PRnet since PRnet outperforms ChemCPA. As illustrated in Figure 2B, our proposed method consistently outperforms PRnet across all three splits. Consistent with results in Figure 2A, it is observed that CatCrossDiT performs more effectively on metrics related to unseen cell line splits, finer grouping metrics, and transcriptome reconstruction, whereas CrossDiT demonstrates superior performance on metrics associated with unseen drugs, coarser grouping metrics, and prediction of transcriptional changes.

These results collectively highlight the superiority of our proposed methods over existing approaches. CatCrossDiT exhibits enhanced integration of transcriptome conditions and improved modeling of perturbation results for individual samples, rendering it more suitable for personalized medicine applications. Conversely, CrossDiT demonstrates superior integration of perturbation conditions and improved identification of consistent perturbation effects on transcriptome, making it more appropriate for new drug discovery scenarios.

To further assess the model’s generalization beyond cell lines and drugs, we extended our analysis to organ‐specific toxicity profiles, a critical dimension for clinical applications. The L1000 dataset is partitioned into three organ‐specific test sets—lung (104,264 samples), kidney (57,304 samples), and pancreas (16,829 samples)—along with a shared training set (500,742 samples) and validation set (166,913 samples). Comparative results with PRnet demonstrate that our method outperforms PRnet across all three organs, highlighting its robust generalization capability to organ‐specific perturbation patterns (Figure S2).

Together, these results establish the framework’s versatility across multiple axes—from molecular (drugs) to cellular (cell lines) to anatomical (organs)—positioning it as a broadly applicable tool for preclinical perturbation modeling.

### 2.2 Evaluation of fusion modules

To verify the rationality and effectiveness of our model structure, we compare a series of model variants (Table 1, methods) to fully demonstrate that the perturbation representation, perturbation fusion module, and transcriptome fusion module we adopt can all improve the final performance of the model.

**TABLE 1:** Model variants of perturbation diffusion transformer (PertDiT).

Model	Perturbation representation	Perturbation fusion module	Transcriptome fusion module
CrossDiT	Text	Cross‐attention	Self‐attention and cross‐attention
CatCrossDiT	Text	Cross‐attention	Concatenation and self‐attention
AdaDiT‐text	Text (averaged)	AdaLN [13]	Concatenation and self‐attention
AdaDiT‐RDKit	RDKit [21]	AdaLN	Concatenation and self‐attention
CatonlyCrossDiT	Text	Cross‐attention	Concatenation

#### 2.2.1 Evaluation of perturbation fusion

To validate the efficacy of our proposed text embedding and cross‐attention modules implemented in CrossDiT and CatCrossDiT, we additionally design a comparison model, AdaDiT, which differs from CatCrossDiT only in its replacement of cross‐attention with adaptive layer normalization AdaLN (Table 1, Methods).

**FIGURE 3:**
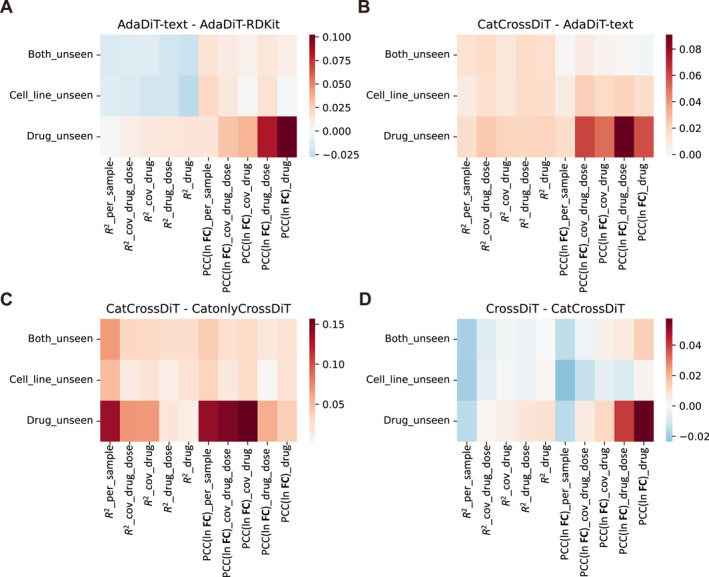
Evaluation of fusion modules. Each figure presents comparative results of the two structures across 10 metrics and three splits. In instances where the title is denoted as Structure1—Structure2, positive values (indicated in red) signify better performance of Structure1, while negative values (indicated in blue) represent worse performance. Performance comparisons are as follows: (A) text‐embedding input versus RDKit input. (B) Cross‐attention versus AdaLN. (C) Presence versus absence of self‐attention. (D) Self‐attention + cross‐attention versus concatenation + self‐attention.

As demonstrated in Figure 3A, text embedding performs better for unseen drugs, and its predicted transcriptional changes are more accurate, indicating that text‐based perturbation modeling is more effective than RDKit. Figure 3B demonstrates that cross‐attention outperforms AdaLN, suggesting that text embedding modeled by cross‐attention retains more comprehensive perturbation information.

To further assess the impact of dosage information on model performance, we conducted dosage ablation experiments. Figure S3 shows the performance of the CrossDiT and CatCrossDiT models without incorporating dosage information during prediction (denoted as CrossDiT_wo_dosage and CatCrossDiT_wo_dosage, respectively). Notably, even in the absence of dosage data, our approach consistently outperforms PRnet across unseen cell lines and both unseen splits (unseen drugs and unseen cell lines). This highlights that when predicting drugs present in the training set, our method can still achieve robust performance by leveraging the intrinsic patterns in drug‐text and transcriptome features. Additionally, the simplified models without dosage information surpass PRnet in per‐sample prediction metrics, underscoring the critical role of the diffusion model’s generative power and Transformer architecture in capturing complex perturbation–transcriptome relationships, even with reduced input features. These results collectively validate the robustness of our core framework and the complementary value of dosage information as an optional enhancement rather than a dependency for basic predictive capability.

#### 2.2.2 Evaluation of transcriptome fusion

In this section, we compare three pre‐perturbation transcriptome fusion strategies: direct concatenation (CatonlyCrossDiT), concatenation followed by self‐attention (CatCrossDiT), and direct cross‐attention (CrossDiT). Figure 3C illustrates that the overall prediction efficacy is substantially enhanced upon the implementation of self‐attention. Figure 3D illustrates that transcriptome fusion with concatenation and self‐attention can improve the transcriptome reconstruction capability for unseen cell lines, as well as the per‐sample metrics, compared with the direct application of cross‐attention. This improvement can be ascribed to the fact that the concatenation operation and MLP are capable of directly modeling the disparities in gene expression profiles between the pre‐ and post‐perturbation states. These disparities may be more beneficial for the final post‐perturbation transcriptome reconstruction, and the strong self‐attention mechanism can also better distinguish between different input transcriptomes, thereby improving the results for single samples. Overall, the strategy of employing concatenation self‐attention used in CatCrossDiT realizes more effective integration of transcriptome information, whereas CrossDiT achieves more accurate prediction of perturbation‐induced changes in gene expression profiles.

### 2.3 Case study

**FIGURE 4:**
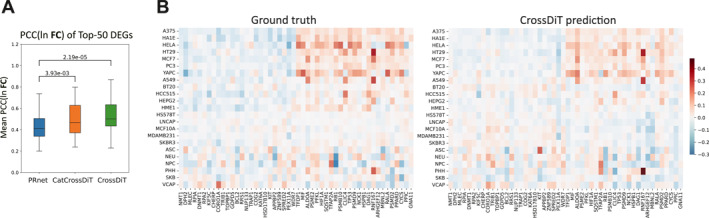
Assessment of predictive accuracy for changes in differentially expressed genes (DEG). (A) Box plots represent the mean PCC(ln **FC**) of the top 50 DEG. *p*‐values are determined using paired *t*‐tests. (B) The heatmaps illustrate the average PCC(ln **FC**) of the gene expression profiles of ground truth and those predicted by CrossDiT under the drug Lapatinib.

Differentially expressed genes (DEG) tend to provide a more representative characterization of drug effects on the transcriptome. We select 25 up‐ and down‐regulated DEGs for each of the top 100 drugs with the largest number of samples. Subsequently, we calculate the mean PCC(ln **FC**) of individual DEGs for each drug and determine that our method significantly outperforms PRnet (Figure 4A).

To further validate the consistency of our model’s predictive performance at the single‐gene level, we expanded the analysis to include all 100 drugs and 978 genes in the dataset. Table S1 provides a comprehensive tabulation of PCC(ln **FC**) values for individual genes across these drugs, showcasing the robustness of our method in capturing gene‐specific perturbation responses. Notably, for each drug‐gene pair, the PCC(ln **FC**) was calculated using all samples perturbed by the corresponding drug.

Additionally, Figure S4 presents boxplots highlighting the top 10 genes with the highest mean PCC(ln **FC**) values among the 100 drugs. These genes exhibit exceptionally strong correlation coefficients, with all top 10 mean values exceeding 0.6. This result provides a multi‐faceted validation of our method’s performance at the single‐gene resolution, reinforcing its utility in capturing fine‐grained drug‐perturbation dynamics.

We then select a representative anticancer drug, Lapatinib, and find that our method is effective in predicting the drug response of various cell lines (Figure 4B). For instance, the upregulated gene changes are more pronounced in VAPC and HELA cell lines, whereas certain cell lines, such as HS578T and LNCAP, exhibit a week response. Taking gene *RNF167* as an example, our model accurately predicts that this gene is most significantly upregulated in HT29, MCF7, A549, and PHH cell lines after Lapatinib perturbation. Conversely, the generally upregulated DEG *RB1* is accurately predicted to be downregulated in ASC, NEU, NPC, and PHH cell lines. Three more cases (SJB‐shh‐31, barasertib‐HQPA, and PLX‐4720) with high prediction correlations can be found in Figure S5. In summary, our model demonstrates the capability to accurately capture the differential effects of various drugs on distinct cell lines.

## 3 DISCUSSION

In the preceding sections, it is demonstrated that our proposed PertDiT model outperforms existing methods. The two designed structures, CrossDiT and CatCrossDiT, are applicable in drug development and personalized medicine contexts, respectively. Through a comprehensive set of well‐designed metrics and an effective data splitting strategy, the effectiveness of our proposed network structure is verified.

Nevertheless, several aspects necessitate further exploration and amelioration. To enhance the model’s inference speed, the adoption of EDM [22] frameworks and consistency models [23] or other optimized sampling methods is contemplated. This will improve computational efficiency and enable faster and more accurate predictions, which are crucial in practical applications.

Our framework is inherently scalable to incorporate diverse textual features and accommodate broader perturbation modeling scenarios. In future work, we plan to integrate richer drug metadata—such as therapeutic indications, mechanistic annotations, and target profiles—to enhance representational power, aligning with advancements in structured biomedical knowledge. Concurrently, the model holds potential for extension to more complex perturbation types, including gene knockouts and protein molecule perturbations, which will broaden its application scope and facilitate a more comprehensive understanding of biological system dynamics.

Building on this scalability, improving model interpretability is equally critical for decoding the relationship between drugs and gene expression profiles. Although our method employs attention mechanisms, their utility is complicated by the multi‐layer architecture, the diffusion process’s focus on predicting noise (rather than direct transcriptomes), and the lack of explicit connections between text tokens and drug structures. To address this, we will leverage modular drug text descriptions alongside simplified network designs or mask‐based ablation experiments, enabling systematic dissection of how different metadata components influence perturbed transcriptome predictions. This dual focus on expanding applicability and enhancing interpretability will solidify PertDiT’s utility as a robust biologically insightful tool for translational research.

Regarding the current limitation of the gene number, which may not fully meet the requirements of perturbation modeling for single‐cell and bulk transcriptome data, we aim to incorporate efficient foundation models such as flash attention [24] and Mamba [25]. By doing so, the input gene numbers are expected to increase, improving both the efficiency and performance of the model. This enhancement will enable the capture of more detailed and accurate information about transcriptional changes under perturbations.

Finally, considering the growing availability of single‐cell perturbation data, our model will be expanded to the single‐cell level in future studies. Adopting an autoencoder combined with diffusion is anticipated to be beneficial in addressing the issue of high dropout in single‐cell data. This approach will likely enhance the model’s robustness and accuracy at the single‐cell level, providing deeper insights into cellular responses to perturbations.

Overall, with the increasing amount of drug‐perturbed transcriptome data and the improvement of more model details, we believe that the accuracy of our model can be further enhanced, and it will be better applied in drug discovery and personalized medicine.

## 4 MATERIALS AND METHODS

### 4.1 Datasets

The L1000 dataset acts as a comprehensive repository of transcriptional perturbation responses. It contains over 1 million bulk RNA‐seq observations with 978 landmark genes. Our preprocessing steps are in line with the PRnet paper. This includes assigning a control sample to each perturbed sample, removing invalid compound SMILES, and filtering compounds with insufficient observations (less than 5). Ultimately, a total of 883,269 observations covering 17,202 compounds and 82 cell lines are obtained.

### 4.2 Experimental setup

We adopt two data splitting modes. One is consistent with PRnet. This splitting includes three rounds of five‐fold cross‐validation, which divides the dataset into approximately 6:2:2 through random splitting, cell line splitting, and drug splitting. The other splitting way is to split SMILES and cell lines separately. After that, five parts can be generated, namely, the training set, validation set, and three test sets: Drug_unseen, Cell_line_unseen, and Both_unseen. Such splitting does not require cumbersome cross‐validation. Models can be trained only once to check the effect on the three types of test sets and can be regarded as a benchmark for this problem.

All data processing is accomplished through the utilization of the Python package Scanpy [26]. Log‐normalization is applied to all gene expression profiles. Subsequently, the DEG for each drug and cell combination are computed. The input to the model consists of a paired control transcriptome, regarded as the pre‐perturbation state, along with the chemical perturbation representation. The output of the model is the predicted post‐perturbation transcriptomes.

### 4.3 Evaluation metrics

We design 10 measures to comprehensively evaluate the prediction performance of the model. These measures are combinations of two metrics and five grouping strategies. The two metrics are the coefficient of determination (*R* ^2^) between the predicted transcriptome and the real transcriptome, and the Pearson correlation coefficient of the natural logarithm of fold change (PCC(ln **FC**)) of the predicted transcriptome compared to the real transcriptome with respect to the control. The calculation of *R* ^2^ and PCC(ln **FC**) on single observation is as follows:

(1) $$\begin{array}{c} R^{2}=R^{2}\left(y_{\text{true}},y_{\text{pred}}\right)=1-\frac{\sum_{1}^{n}{\left(y_{{\text{tru}e}_{i}}-y_{{\text{pre}d}_{i}}\right)}^{2}}{\sum_{1}^{n}{\left(y_{{\text{tru}e}_{i}}-{\bar{y}}_{{\text{pre}d}_{i}}\right)}^{2}} \end{array}$$

(2) $$\begin{array}{c} {\ln\text{FC}}_{\text{true}}=y_{\text{true}}-x_{\text{control}} \end{array}$$

(3) $$\begin{array}{c} {\ln\text{FC}}_{\text{pred}}=y_{\text{pred}}-x_{\text{control}} \end{array}$$

(4) $$\begin{array}{l} \text{PCC}\left(\ln\text{FC}\right)=\text{PCC}\left({\ln\text{FC}}_{\text{true}},{\ln\text{FC}}_{\text{pred}}\right)\\ =\frac{\sum_{1}^{n}\left({\ln\text{FC}}_{{\text{tru}e}_{i}}-\bar{{\ln\text{FC}}_{\text{true}}}\right)\left({\ln\text{FC}}_{{\text{pre}d}_{i}}-\bar{{\ln\text{FC}}_{\text{pred}}}\right)}{\sigma_{{\;\ln\text{FC}}_{\text{true}}}\sigma_{{\;\ln\text{FC}}_{\text{pred}}}} \end{array}$$

The five grouping strategies from coarse to fine as follows: those treated with the same drug (drug), the same drug and dose (drug_dose), the same drug and the same cell (cov_drug), the same drug, cell, and dose (cov_drug_dose) and no grouping (per_sample). After grouping, the expression levels within each group are averaged, and then the fold change and correlation coefficients are calculated. For example, if we can obtain *n* groups grouped by cov_drug_dose, two metrics can be calculated as follows:

(5) $$\begin{array}{c} {y_{\text{true}}}_{\text{Group}i}=\underset{j\in \text{Group}i}{\text{Mean}}{y_{\text{true}}}_{j} \end{array}$$

(6) $$\begin{array}{c} {y_{\text{pred}}}_{\text{Group}i}=\underset{j\in \text{Group}i}{\text{Mean}}{y_{\text{pred}}}_{j} \end{array}$$

(7) $$\begin{array}{c} {x_{\text{control}}}_{\text{Group}i}=\underset{j\in \text{Group}i}{\text{Mean}}{x_{\text{control}}}_{j} \end{array}$$

(8) $$\begin{array}{c} {\ln\text{FC}}_{{\text{true}}_{\text{Group}i}}={y_{\text{true}}}_{\text{Group}i}-{x_{\text{control}}}_{\text{Group}i} \end{array}$$

(9) $$\begin{array}{c} {\ln\text{FC}}_{{\text{pred}}_{\text{Group}i}}={y_{\text{pred}}}_{\text{Group}i}-{x_{\text{control}}}_{\text{Group}i} \end{array}$$

(10) $$\begin{array}{c} R_{\text{Group}i}^{2}=R^{2}\left({y_{\text{true}}}_{\text{Group}i},{y_{\text{pred}}}_{\text{Group}i}\right) \end{array}$$

(11) $$\begin{array}{c} {\text{PCC}}_{\text{Group}i}\left(\ln\text{FC}\right)=\text{PCC}\left({\ln\text{FC}}_{{\text{true}}_{\text{Group}i}},{\ln\text{FC}}_{{\text{pred}}_{\text{Group}i}}\right) \end{array}$$

(12) $$\begin{array}{c} R_{\text{cov}\_\text{drug}\_\text{dose}}^{2}=\frac{1}{n}\sum_{i=1}^{n}R_{\text{Group}i}^{2} \end{array}$$

(13) $$\begin{array}{c} \text{PCC}{\left(\ln\text{FC}\right)}_{\text{cov}\_\text{drug}\_\text{dose}}=\frac{1}{n}\sum_{i=1}^{n}{\text{PCC}}_{\text{Group}i}\left(\ln\text{FC}\right) \end{array}$$

### 4.4 Multi‐conditional diffusion model

In this study, we denote the perturbed transcriptome as ***x***_0_, the pre‐perturbed transcriptome as ***c***_pre_, and the perturbation as ***c***_pert_. Unconditional DDPM learn a distribution *p*_*θ*_(***x***_0_) to fit the real data distribution *q*(***x***_0_) by gradually adding noise in the forward process and denoising from pure Gaussian noise ***x***_T_ in *T* steps in the reverse process.

Similar to the practice in the CSDI [27] model, we can naturally extend the DDPM into a multi‐conditional model, enabling the conditions to control the diffusion model to generate the transcriptomes of specific cells perturbed by specific perturbations.

Our multi‐conditional diffusion model PertDiT estimates the real conditional distribution of perturbed transcriptome *q*(***x***_0_| ***c***_pre_, ***c***_pert_) with a learned model distribution *p*_*θ*_(***x***_0_| ***c***_pre_, ***c***_pert_). We set the noise variance schedule as follows:

(14) $$\begin{array}{c} \beta_{1}<\beta_{2}<\text{…}<\beta_{T},\lim_{T\to \infty }\beta_{T}=1,\alpha_{t}=1-\beta_{t},{\bar{\alpha }}_{t}=\prod_{i=1}^{t}\alpha_{i} \end{array}$$

Let us denote ***x***_*t*_ as the noisy data generated by adding noise *t* times on the real data ***x***_0_ and the forward process is as follows:

(15) $$\begin{array}{c} x_{t}=\sqrt{{\bar{\alpha }}_{t}}x_{0}+\sqrt{{1-\bar{\alpha }}_{t}}\epsilon ,\epsilon ∼N\left(0,I\right) \end{array}$$

***ϵ***_*θ*_ is the learnable noise predictor of PertDiT and the reverse process controlled by conditions is as follows:

(16) $$\begin{array}{c} x_{t-1}=\frac{1}{\sqrt{\alpha_{t}}}\left(x_{t}-\frac{\beta_{t}}{\sqrt{1-{\bar{\alpha }}_{t}}}\epsilon_{\theta }\left(x_{t},t|c_{\text{pre}},c_{\text{pert}}\right)\right)+\sigma_{t}z \end{array}$$

(17) $$\begin{array}{c} \sigma_{t}=\sqrt{\frac{1-{\bar{\alpha }}_{t-1}}{1-{\bar{\alpha }}_{t}}\cdot \beta_{t\;}},z∼N\left(0,I\right) \end{array}$$

During training, we randomly sample noisy ***x***_*t*_ and train ***ϵ***_*θ*_ by minimizing the following loss:

(18) $$\begin{array}{cc} \min_{\theta }\;L\left(\theta \right)∶ & =\min_{\theta }E_{x_{0}∼q\left(x_{0}|c_{\text{pre}},c_{\text{pert}}\right),\epsilon ∼N\left(0,I\right),t}\\ & {\left\|\epsilon -\epsilon_{\theta }\left(x_{t},t|c_{\text{pre}},c_{\text{pert}}\right)\right\|}^{2} \end{array}$$

When generating, we sample ***x***_T_ ∼ *N*(0, ***I***) and iteratively denoise it *T* times following the reverse process to get the predicted ***x***_0_.

### 4.5 Perturbation representation

Considering that the representations of perturbations encompass not only small molecules but also proteins, gene knockouts, treatment methods, and so on, we have adopted a unified perturbation representation method based on text representation to enhance the extensibility of perturbation modeling. In this paper, we primarily focus on modeling small molecules. Firstly, we transform the SMILES of small molecules into standard text descriptions by utilizing the pre‐trained MolT5 model. In addition to molecular information, the L1000 data perturbations also involve small molecule dosage information. Hence, we append a prompt, “The dosage is [] micromoles” after the text description of the small molecule. Finally, we convert the entire text description of the molecule and dosage into a representation ${\text{Emb}}_{\text{text}}\in R^{n_{\text{token}}\times d_{\text{text}}}$ by utilizing the pre‐trained Linkbert model. To adapt to AdaLN or reduce computational complexity, it is also possible to obtain the average‐pooling vector representation ${\text{Emb}}_{\text{text}}\in R^{d_{\text{text}}}$.

Regarding the RDKit embedding employed in PRnet, the fixed‐size fingerprint embedding (FCFP4 fingerprints) of a SMILES is generated by the RDKit encoder. The final RDKit embedding is the fingerprint embedding weighted by the log_10_‐scaled dosage value.

### 4.6 Network architecture

PertDiT consists of two parts: adapters and transformer layers. We propose two architectures of transformer layers, namely, CrossDiT and CatCrossDiT.

The adapters are all single‐layered linear layers, with the aim of mapping inputs of different dimensions to the same dimension to facilitate the processing by the transformer layers. Specifically, this is achieved by the following three types of linear layers: Pert‐adapter: $R^{n_{\text{token}}\times d_{\text{text}}}\to R^{n_{\text{token}}\times d_{\text{model}}}$, trans‐adapter: $R^{n_{\text{gene}}}\to R^{n_{\text{gene}}\times d_{\text{model}}}$, and T‐adapter: $R\to R^{d_{\text{model}}}$. A slight difference lies in the handling of transcriptome data ***x***_*t*_ and ***c***_pre_: in CrossDiT, two linear layers are used to process ***x***_*t*_ and ***c***_pre_ separately, whereas in CatCrossDiT, ***x***_*t*_ and ***c***_pre_ are concatenated first and then processed by a single linear layer ($R^{n_{\text{gene}}\times 2}\to R^{n_{\text{gene}}\times d_{\text{model}}}$).

After being processed by the adapters, ***x***_*t*_, ***c***_pre_, and ***c***_pert_ all need to be added to the expanded time embedding *t*. In addition, the transcriptome inputs ***x***_*t*_ and ***c***_pre_ also require the learnable gene representations ${\text{Pos}\in R}^{n_{\text{gene}}\times d_{\text{model}}}$ to be added to them before being input into the transformer layer.

The transformer layers all adopt the pre‐norm residual structure [28]. CrossDiT consists of two cross‐attention modules which are used to fuse ***x***_*t*_ with ***c***_pre_ and ***c***_pert_ in sequence, and a feed‐forward module realized by a two‐layer MLP. CatCrossDiT consists of a self‐attention module, a cross‐attention module which is used to fuse ***c***_pert_ and ***x***_*t*_, and a feed‐forward module same as CrossDiT. The cross‐attention module can be represented as follows:

(19) $$\begin{array}{c} Q=W_{Q}x^{T} \end{array}$$

(20) $$\begin{array}{c} K=W_{K}c^{T} \end{array}$$

(21) $$\begin{array}{c} V=W_{V}c^{T} \end{array}$$

(22) $$\begin{array}{c} \text{Attention}\left(x,c\right)=\text{softmax}\left(\frac{Q^{T}K}{\sqrt{d}}\right)V^{T} \end{array}$$

The self‐attention module can be represented by Attention(*x*, *x*).

We also additionally designed an AdaDiT to compare the effects of perturbation modeling. The only difference between AdaDiT and CatCrossDiT is that the cross‐attention layer is replaced by adaptive layer normalization, which is more suitable for vector‐like inputs. The specific formulas are as follows:

(23) $$\begin{array}{c} \alpha ,\beta ,\gamma =\text{MLP}\left(c_{\text{pert}}\right) \end{array}$$

(24) $$\begin{array}{c} x_{\text{scale}\&\text{shift}}=\gamma ⊙x+\beta \end{array}$$

(25) $$\begin{array}{c} x_{\text{gate}}=\alpha ⊙x \end{array}$$

Each layer of AdaDiT has two sets of (*α*, *β*, *γ*). The scale&shift operations are positioned prior to the self‐attention and feed‐forward modules, whereas the paired gate operations are located after the self‐attention and feed‐forward modules.

## AUTHOR CONTRIBUTIONS

**Qifan Hu**: Conceptualization; data curation; methodology; software; validation; visualization; writing—original draft. **Zeyu Chen**: Writing—review and editing. **Jin Gu**: Funding acquisition; supervision; writing—review and editing.

## CONFLICT OF INTEREST STATEMENT

The authors declare no conflicts of interest.

## ETHICS STATEMENT

None of the authors of this article have conducted any studies involving human or animal subjects.

## Supporting information

Supporting Information S1

Supporting Information S2

Table S1

## Data Availability

All codes and data for PertDiT are available at GitHub website (wangkekekeke/PertDiT.git).
