# Supplementary material for: Predicting drug‐perturbed transcriptional responses using multi‐conditional diffusion transformer
Source: Quant Biol. 2025 Sep 21;14(1):e70016. doi: 10.1002/qub2.70016 (PMC12806128; doi:10.1002/qub2.70016)
Supplement: Supplementary file 1 — Supporting Information S1 [file QUB2-14-e70016-s003.docx]

# Supporting Figure


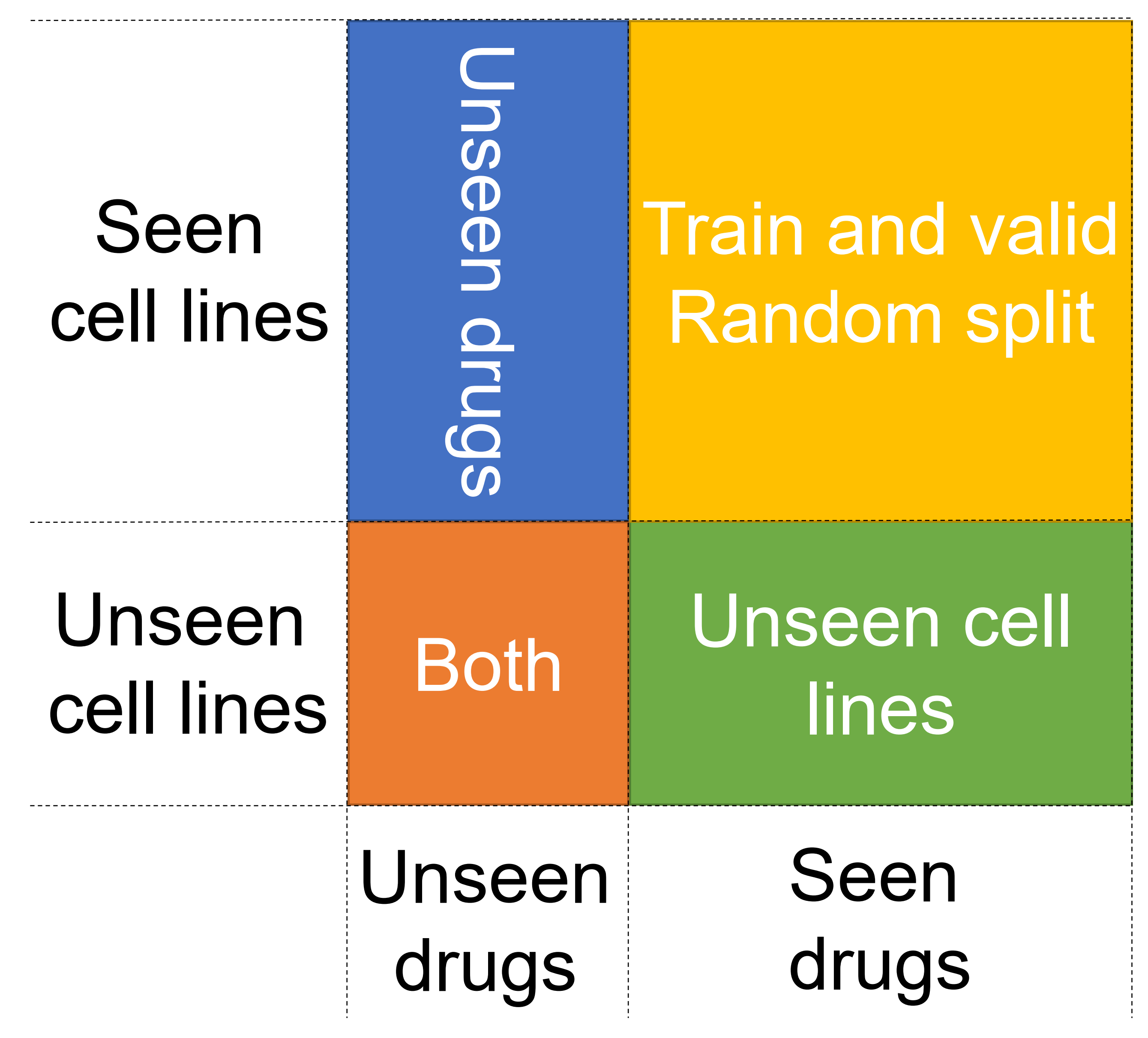


**Figure S1:** Schematic diagram of our proposed one-time splitting strategy.


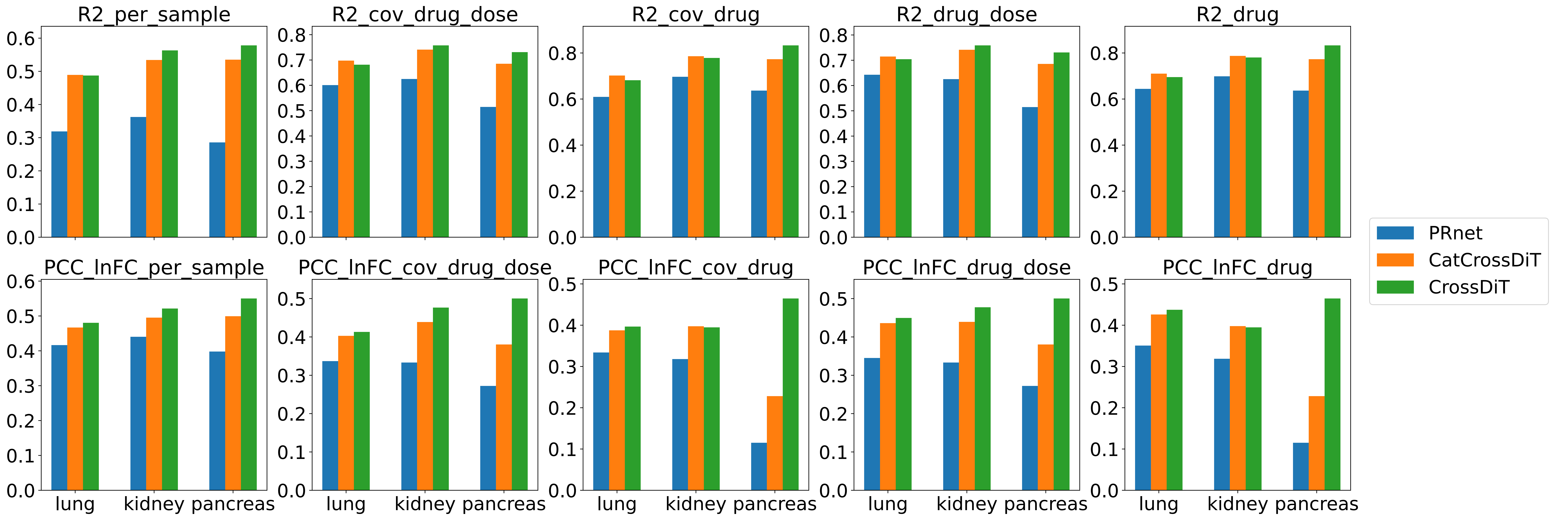


**Figure S2** Performance comparison of out-of-distribution transcriptome prediction in unseen organ splits. Ten metrics and three test sets (lung, kidney and pancreas) are shown by bar plots.


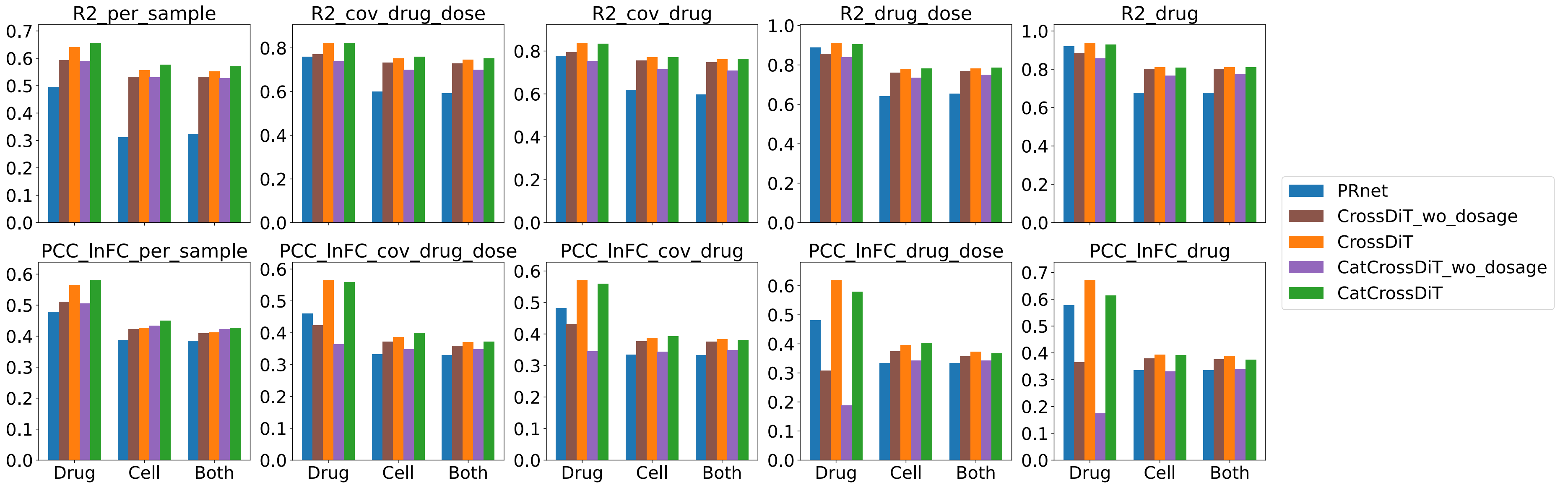


**Figure S3** Performance comparison of out-of-distribution transcriptome prediction in dose ablation experiments. Ten metrics and three splitting strategies same as Figure 2A are shown by bar plots. Five methods are compared: PRnet, CrossDiT with or without dosage (CrossDiT_wo_dosage) and CatCrossDiT with or without dosage (CatCrossDiT_wo_dosage).


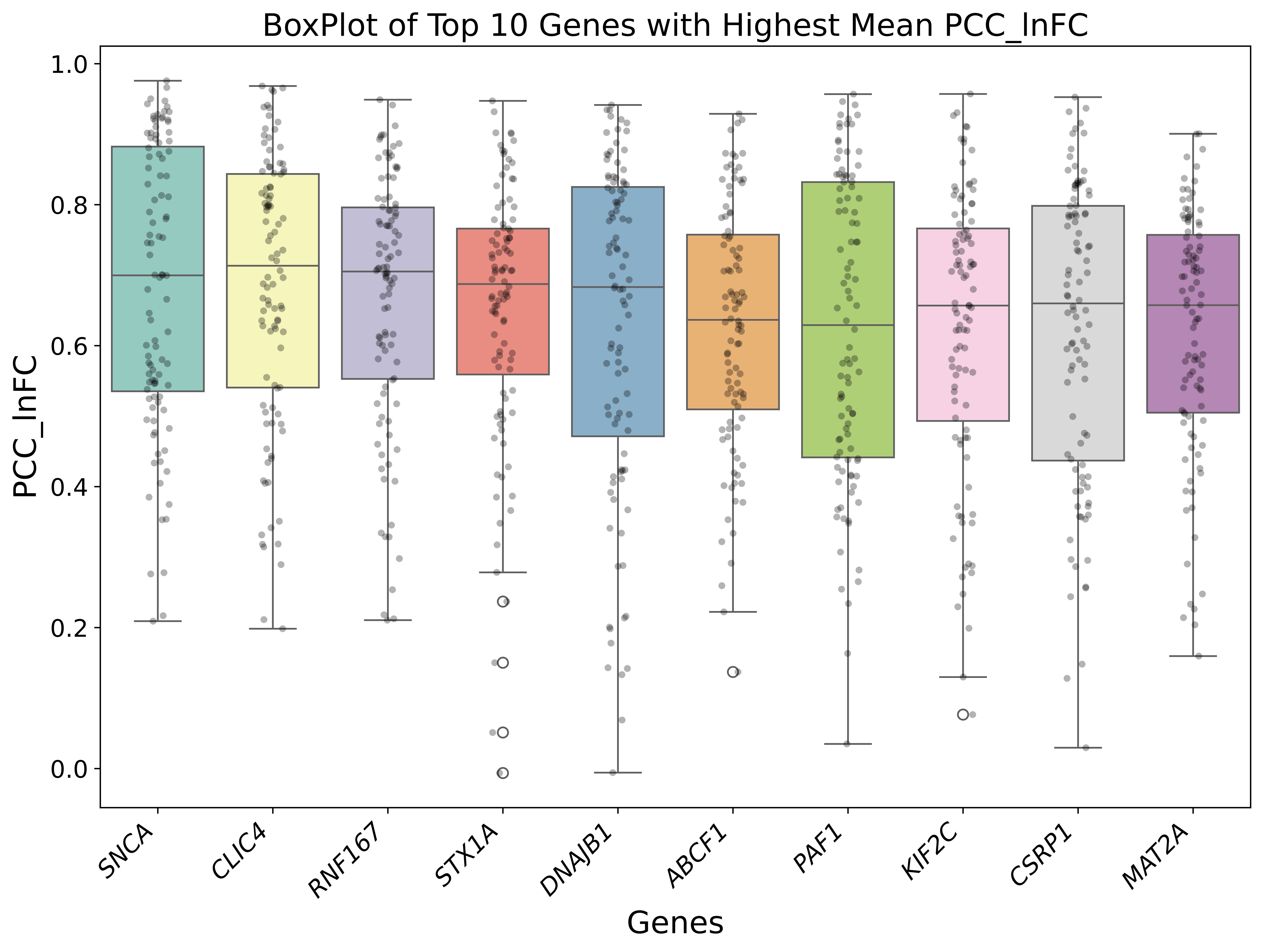


**Figure S4** Boxplots of top 10 genes with highest mean $PCC\left( \ln\boldsymbol{FC} \right)$ among the top 100 drugs with the largest number of samples.


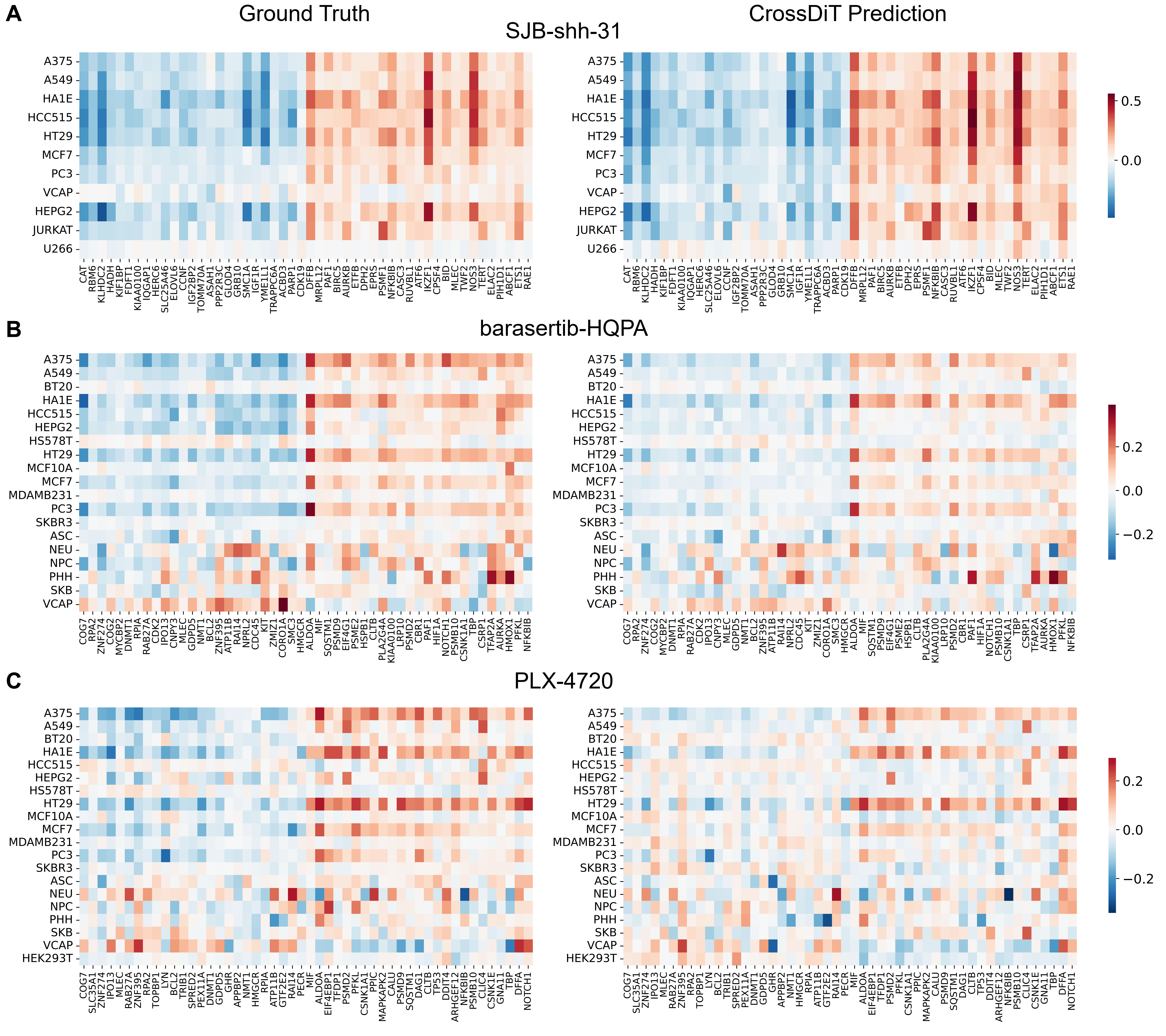


**Figure S5** Three cases of assessment of predictive accuracy for changes in differentially expressed genes.
