# Supplementary material for: Predicting drug‐perturbed transcriptional responses using multi‐conditional diffusion transformer
Source: Quant Biol. 2025 Sep 21;14(1):e70016. doi: 10.1002/qub2.70016 (PMC12806128; doi:10.1002/qub2.70016)
Supplement: Supplementary file 2 — Supporting Information S2 [file QUB2-14-e70016-s001.docx]

PCC_lnFC for individual genes of the top 100 drugs with the largest number of samples
